# Transcriptome profiling of developmental and xenobiotic responses in a keystone soil animal, the oligochaete annelid *Lumbricus rubellus*

**DOI:** 10.1186/1471-2164-9-266

**Published:** 2008-06-03

**Authors:** Jennifer Owen, B Ann Hedley, Claus Svendsen, Jodie Wren, Martijs J Jonker, Peter K Hankard, Linsey J Lister, Stephen R Stürzenbaum, A John Morgan, David J Spurgeon, Mark L Blaxter, Peter Kille

**Affiliations:** 1 Cardiff School of Biosciences, BIOSI 1, University of Cardiff, P.O. Box 915, Cardiff, CF10 3TL, UK; 2 Institute of Evolutionary Biology, University of Edinburgh, King's Buildings, Edinburgh, EH9 3JT, UK; 3 Centre for Ecology and Hydrology, Monks Wood, Abbots Ripton, Huntingdon, Cambridgeshire, PE28 2LS, UK; 4 Microarray Department and Integrative Bioinformatics Unit, Faculty of Science, University of Amsterdam, Kruislaan 318, Building I, Room 105C, 1098 SM Amsterdam, The Netherlands; 5 School of Biomedical and Health Sciences, Pharmaceutical Sciences Division, Franklin Wilkins Building 150, Stamford St., London, SE1 9NH, UK

## Abstract

**Background:**

Natural contamination and anthropogenic pollution of soils are likely to be major determinants of functioning and survival of keystone invertebrate taxa. Soil animals will have both evolutionary adaptation and genetically programmed responses to these toxic chemicals, but mechanistic understanding of such is sparse. The clitellate annelid *Lumbricus rubellus *is a model organism for soil health testing, but genetic data have been lacking.

**Results:**

We generated a 17,000 sequence expressed sequence tag dataset, defining ~8,100 different putative genes, and built an 8,000-element transcriptome microarray for *L. rubellus*. Strikingly, less than half the putative genes (43%) were assigned annotations from the gene ontology (GO) system; this reflects the phylogenetic uniqueness of earthworms compared to the well-annotated model animals. The microarray was used to identify adult- and juvenile-specific transcript profiles in untreated animals and to determine dose-response transcription profiles following exposure to three xenobiotics from different chemical classes: inorganic (the metal cadmium), organic (the polycyclic aromatic hydrocarbon fluoranthene), and agrochemical (the herbicide atrazine). Analysis of these profiles revealed compound-specific fingerprints which identify the molecular responses of this annelid to each contaminant. The data and analyses are available in an integrated database, LumbriBASE.

**Conclusion:**

*L. rubellus *has a complex response to contaminant exposure, but this can be efficiently analysed using molecular methods, revealing unique response profiles for different classes of effector. These profiles may assist in the development of novel monitoring or bioremediation protocols, as well as in understanding the ecosystem effects of exposure.

## Background

Ever since Charles Darwin's classic work [1], earthworms (Phylum Annelida, Class Oligochaeta) have been renowned as 'ecosystem engineers' in recognition of the direct and indirect effects they have on water, nutrient and carbon cycling in temperate and tropical soils [2]. Earthworms have therefore been widely adopted by international and national agencies for the diagnosis of soil ecosystem health, and for predicting the potential environmental impact of xenobiotics, such as industrial chemicals, pesticides and medicines, from anthropogenic sources [3]. Quantification of chemical toxicity to earthworms currently relies on measuring the effects of exposure on key life-history traits (survival, growth, and reproduction) in standardised laboratory bioassays conducted with certain test species (*Eisenia fetida*, *Eisenia andrei *and *Lumbricus rubellus*). These bioassays can produce sensitive estimates of population effects, but are not suited to elucidation of mechanisms of action, and thus may be difficult to generalise from. For example, xenobiotic exposure may affect individual physiology, cocoon-lay rates, cocoon viability and juvenile growth rates in a specific manner, resulting in different outcomes for population growth rate and age structure.

Complementing measurements of gross toxicity with molecular profiling and genomic studies can make plain the modes of action of specific xenobiotics and identify the generality of biological process affected as well as the molecular response pathways invoked [4-8]. Taking a specific example, it has been observed that *L. rubellus *is able to colonise highly metal-contaminated environments [8,9]. Detailed work identified that the primary molecules responsible for this metal tolerance were earthworm metallothioneins [10-15]. Though successful, the progress of these mechanistic studies has been handicapped by the lack of available sequence data for earthworms in public databases and the finding that very few (<30%) of the few available earthworm gene fragments could be identified by sequence similarity to previously sequenced genes.

To help bridge this gap in available sequence information, a previously described small-scale (600 sequences) expressed sequence tag (EST) dataset for *L. rubellus *was generated [16]. This has prompted the extended survey of the *L. rubellus *transcribed genome through extensive sampling of additional cDNA libraries from earthworms at defined lifecycle stages (late embryo, juvenile, adult), specific tissues (anterior segments and reproductive organs) and following acute exposure to model chemicals representing three different contaminant classes: inorganic (the nonessential heavy metal cadmium [Cd] and the essential metal copper [Cu]), organic (the polycyclic aromatic hydrocarbon [PAH], fluoranthene [FLA]), and agrochemical (the herbicide atrazine [ATZ]) that is reported here. From this transcriptome resource, we defined ~8100 genes, and have fabricated a cDNA microarray to investigate the transcriptome responses of *L. rubellus *through normal growth and following sub-lethal exposure to a series of sub-lethal concentrations of Cd, FLA, and ATZ. From the derived transcript profiles we identify the key molecular responses and biochemical pathways associated with each life-stage treatment, and propose mechanisms of action for the three chemicals.

## Results and Discussion

### Generation of ESTs from cDNA libraries spanning the *L. rubellus *lifecycle and following exposure to target chemicals

We generated 17,225 high-quality ESTs from nine different cDNA libraries constructed from *L. rubellus *raised under defined conditions (Table 1 and Additional File 1; all sequences are available in EMBL/GenBank/DDBJ and in the project database LumbriBASE [see below]). We maximised the gene discovery rate by screening out, from some libraries, a small number of highly-expressed mRNAs (identified as clusters with many members after initial sequencing of ~4,000 ESTs). The ESTs were clustered using the PartiGene suite of tools [17] and 8,129 clusters (different putative genes, named using permanent identifiers of the form 'LRC#####') were defined. This number of putative genes is likely to be an overestimate because (1) alternative splice forms may be assembled into different clusters, (2) the partial nature of ESTs may result in non-overlap between sequences from the same mRNA, and (3) our earthworms were drawn from an outbred source and allelic variation is likely. Within-dataset analyses suggested that the effects of these factors are minor, and that the true diversity in the dataset is ~8,000 different loci. If, like other non-vertebrates, *L. rubellus *has between 15,000 and 20,000 protein-coding genes, the sequencing effort has generated sequence tags for ~40–50% of all genes in this key species.

**Table 1:** The *Lumbricus rubellus *Expressed Sequence Tag dataset.

**Library**	**Earthworm treatment regime**^1^	**Number of ESTs**	**Mean EST length (bp) ESTs ± SD**	**No. of clusters (putative genes)**^2^	**Mean consensus length (bp)**	**Mean translation length (aa)**	**Redundancy (ESTs/gene)**
1	Adult worms collected from a control field site and acclimatised to test condition in the laboratory	1173	550.4 (± 132.7)	718	759.9 (± 350.7_	195.5 (± 103.5)	1.634
2	Late developmental stage (~5 weeks post laying) embryonic tissue dissected from cocoons laid by paired unexposed adult worms	2728	622.6 (± 85.9)	1314	772.4 (± 291.6)	148.7 (± 95.4)	2.076
3	~40 day post hatch juveniles (at mid log growth phase = approximately 300 mg) reared from hatchling emerging from cocoons laid by paired unexposed adult worms	2859	405.8 (± 125.9)	1757	598.0 (± 328.0)	106.5 (± 84.7)	1.627
4	Head enriched (anterior segments 1–33) from acclimatised adult worms	2569	537.5 (± 173.0)	1357	655.6 (± 301.8)	114.4 (± 89.6)	1.893
5	Mix of tissue from acclimatised adult worms exposed to either 50, 200 or 600 mg Cd/kg dry soil	2230	405.8 (± 125.9)	1548	710.9 (± 291.6)	113.7 (± 84.2)	1.441
6	Mix of tissue from acclimatised adult worms exposed to either 62, 140, 316, 711 and 1066 mg FLA/kg dry soil	2336	566.7 (± 133.1)	1625	732.3 (± 307.6)	145.9 (± 96.8)	1.438
7	Mix of tissue from acclimatised adult worms exposed to either 12 and 35 mg ATZ/kg dry soil	1739	568.2 (± 169.1)	1386	706.0 (± 299.5)	124.5 (± 91.7)	1.255
8	Mix of tissue from acclimatised adult worms exposed to either 40, 160, 460 and 480 mg Cu/kg dry soil	1518	544.0 (± 176.9)	1084	662.0 (± 283.1)	122.3 (± 85.7)	1.400
9	Reproductive organs (following a subtractive hybridisation protocol)	73	321.2 (± 94.9)	66	372.7 (± 212.2)	87.3 (± 58.3)	1.106

Total		17225	539.7 (± 155.0)	8129	611.0 (± 243.0)	107.4 (± 74.9)	2.119

1. See Additional File 1 for more detailed descriptions of the libraries used.

2. A cluster is counted as derived from a library if it contains at least one EST from that library. Thus the longest cluster (LRC00201, encoding paramyosin) contains ESTs from five of the nine libraries and counts as a member of all five.

**Figure 1:**
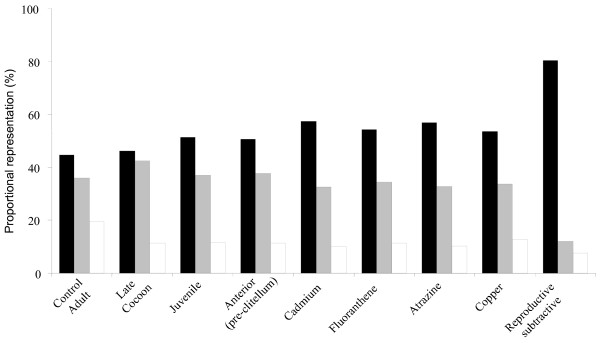
**Proportional representation of ESTs within clusters for sequences determined from each *L. rubellus *cDNA library**. The figure illustrates the proportion of the ESTs generated from each library which are associated within clusters containing a total of 1 (singletons, Black bars), 2–9 (grey bars) and 10 or more ESTs (Clear bars).

Clusters (putative genes) with one EST member accounted for 53% (5361) of all clusters (Figure 1). There were 7913 clusters containing ten or fewer ESTs (97% of the total), and only 20 clusters had over 50 ESTs. Of the clusters with more than one EST, approximately 50% were library-specific. Such specificity may simply reflect the low density of sampling rather than real expression differences between libraries. For each cluster, we derived a consensus cDNA sequence, and from this derived a putative protein translation using prot4EST [18]. Some clusters (281) yielded more than one consensus, possibly due to alternative splice forms or divergent alleles. Across all the *L. rubellus *libraries, the 8,129 consensus sequences had a mean length of 611 bases (± 243) (Table 1). The mean length of *L. rubellus *putative proteins was 107 amino acids (i.e. 53% of the mean consensus length). Variation in mean protein length between libraries was directly related to the variation in consensus length.

The sequence and annotation data (including clustering, consensus sequences, predicted protein sequences, and functional categorisations) are available in a relational database, LumbriBASE. The database has been used as a workbench for our analyses (including microarray experiment annotation and visualisation; see below), and is presented on the web through a PHP-scripted interface [19].

### Functional annotation of the *L. rubellus *gene set

**Figure 2:**
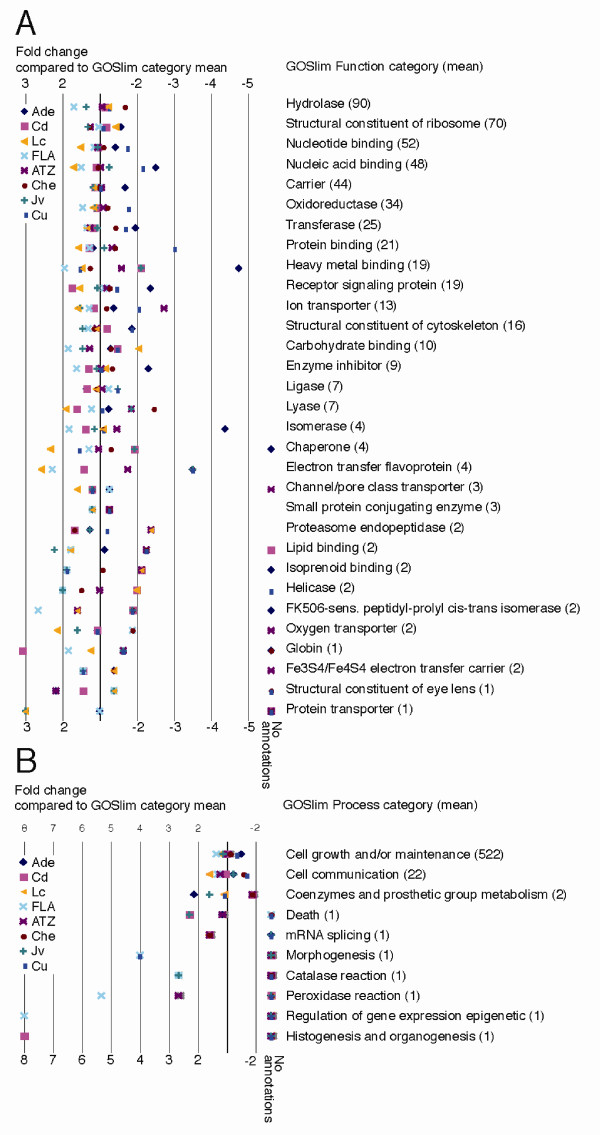
**GO-Slim analysis of annotated ESTs**. The clustered EST data were annotated with terms from the GOSlim subset of the Gene Ontology system. For each term with more than eight clusters annotated across all libraries, we calculated the mean presence of that term per library, and the fold difference, compared to this mean, of the presence in each library. Panel A: GOSlim Function terms. Panel B: GOSlim Process terms. The acronyms for the cDNA libraries are: Ade, control adult earthworms; Lc, control late cocoon stage earthworms; Jv, control juvenile earthworms; Che, control adult earthworms, anterior segments; Cd, cadmium-exposed earthworms; Cu, copper-exposed earthworms; FLA, fluoranthene-exposed earthworms; ATZ, atrazine-exposed earthworms (for details, see Materials and Methods). For each GOslim term, the mean number of terms observed across all libraries is given in brackets. Some libraries had no annotations for particular GOslim terms (grouped as 'no annotations' on the figure).

Gene ontology (GO) terms [20] were assigned to *L. rubellus *predicted proteins using GOtcha [21] and the analysis summarised by assessing representation of higher-level (GOSlim) terms (Figure 2). Less than half (3460; 43%) of the putative protein translations were assigned a GO term at a confidence greater than 50. This apparently high proportion of unannotated genes in *L. rubellus *can be explained by our application of the conservative GOtcha algorithm and by the paucity of genomic information from other annelid species. For those GOSlim "Function" and "Process" categories with a mean representation across all libraries of ≥ 1, we examined under- and over-representation in each library (Figure 2). The developing cocoon library had the highest representation of nucleic acid binding, nucleotide binding, chaperone, and protein binding, likely derived from the presence of actively developing embryos in these animals, while the juvenile library had an over representation of lipid-binding annotations (Figure 2A). As expected, the control adult library was lacking in terms associated with heavy metal binding compared to libraries derived from Cu- or Cd-treated earthworms. However, unexpectedly, the library from FLA-exposed animals also had increased representation of this annotation. The FLA library also exhibited overrepresentation of carbohydrate-binding and FK506-sensitive peptidyl-prolyl isomerase annotations. In the library from Cd-treated animals both receptor signalling and globin classes were abundant compared to other libraries, perhaps reflecting the interference of Cd with the functions of other, essential metals. In the less-closely defined "process" categories, Cd-treated and juvenile worm-derived libraries had more annotations of 'cell death' than other libraries (Figure 2B). Many of these general observations were confirmed through the use of microarrays (see below).

The only other "soil" animal for which extensive gene annotation data exist is the rhabditid nematode *Caenorhabditis elegans*, though ecotoxicology analyses focussed on this species are limited [22]. Identification of possible orthologues between *L. rubellus *and *C. elegans *using BLAST similarity identified 1009 (12%) *L. rubellus *sequences with a BLAST hit in the *C. elegans *proteome (Wormpep 145) with an e-value of less than 1e-25, 569 of which were reciprocal top hits between the species (identified on the protein report pages of LumbriBASE). Of these, 158 had annotations relating to *C. elegans *RNAi experiments (as represented in WormBase [23]) but these were largely uninformative as to biological function.

### Fabrication and validation of a *L. rubellus *cDNA microarray

**Figure 3:**
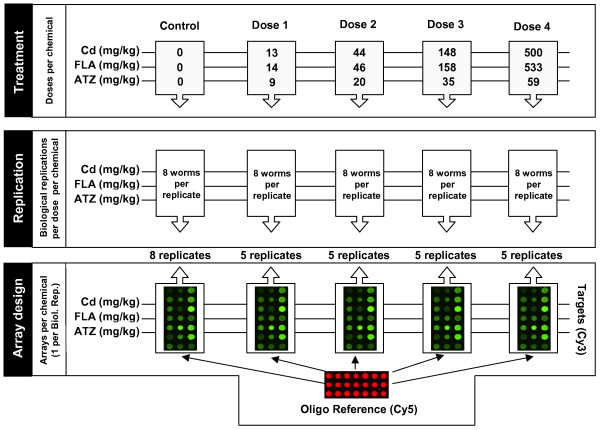
**Schematic representation of the experimental design for xenobiotic exposure**. The figure depicts the 5 doses used for each chemical exposure, together with the number of true (independent) biological replicates, each replicate consisting of 8 (pooled) individual earthworms that have been independently exposed to the relevant treatment condition. Control groups contained 8 replicates, and each of the treatment conditions (i.e. chemical and dose) contained 5 replicates. The design of the array experiment shows the labelling, hybridisation and analysis steps for each biological replication.

To profile life-cycle and xenobiotic transcriptome responses, a custom cDNA microarray was fabricated using representative reporters from each cluster. Each spotted cDNA originated from a clone processed for sequencing. The hybridisation was performed using a nested reference design. In detail, the reference sample (labelled with Cy5) was derived from two 65–70-mer oligonucleotides designed against vector sequence between the amplification primer and the inserted cDNA and co-amplified during insert preparation (Additional File 2). Probes derived from mRNA preparations were labelled with Cy3. This nested reference design permitted direct comparison between samples both within and between experiments. Four experiments were undertaken, comparing juvenile and adult stages and comparing transcriptional responses following exposure to Cd, FLA and ATZ. All exposures were generated using a common experimental set-up designed to generate a set of biologically replicated samples for a series of exposure concentrations (Figure 3).

After hybridisation, array scanning, and pre-processing, the initial technical validation included visual inspection of images to identify gross abnormalities or background. Prior to normalisation the sensitivity of the array and relationship between RNA concentration and fluorescent signal was assessed by calculating the signal intensity generated by reporters complementary to 10 "alien" RNA spikes introduced at known concentrations, from 1 pmole to 30 nmole, prior to labelling (exemplar plots are provided in Additional File 3). Following normalisation the distribution of data responses was examined and any samples showing abnormal distribution were discarded from the analysis (see Additional File 4). Array data were further validated by generating MA-plots, graphical representations of log ratio of the average normalised data from control samples compared to xenobiotic-exposed counterparts against the fluorescence intensity [24] (see Additional File 5). The control samples were of high quality by this measure. For example, when compared to the Cd exposure controls, >99% of the genes exhibiting an acceptable signal had <1.8-fold variance in expression (and >97.5% showed a <1.4-fold variance). In contrast, for hybridisations involving mRNA from animals exposed to 500 μg/kg Cd, ~5% of genes showed an expression change >1.8-fold with respect to the controls (with 15% changing >1.4-fold) (Additional File 5). Further, a group of reporters showed very significant up-regulation in response to Cd. Annotations for these reporters indicated that they included clusters encoding various metallothionein isoforms, a gene set previously shown to be highly Cd responsive (Additional File 5) [13-15].

### Gene expression changes through the *L. rubellus *lifecycle

**Figure 4:**
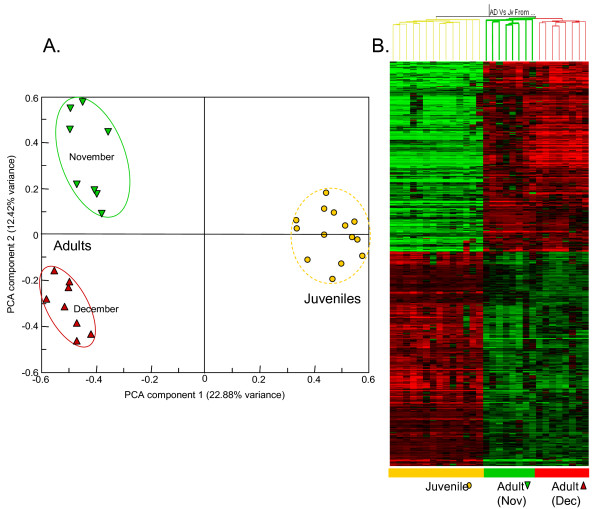
**Analysis of differential transcription between adult and juvenile earthworms**. Panel A presents a Principal Component Analysis of transcript profiles from adult and juvenile earthworms. The array data used for this analysis were normalised using per chip and per gene median polishing, and data from poor quantity spots were removed; no additional filtering was performed. Panel B displays hierarchical clustering (using a distance algorithm for both conditions and genes) for genes showing significant (p < 0.01) differential expression using a t-test with Benjamini and Hochberg False Discovery. Juvenile data are represented as yellow spheres and adult data are shown as triangles (red indicate those organisms sampled in November and green for those harvested in December).

Transcript profiles were compared for 14 control groups of juveniles and biological replicate groups of adults (16 groups). These groups were derived from a common stock and selected to minimise the possible confounding effects of seasonal variation (details of experimental conditions and treatment metadata for each array have been submitted with the array data; for details see ArrayExpress: E-MAXD-36). After initial pre-processing of the data, including normalisation relative to the median gene expression in juveniles and adults combined, both principal component analysis and hierarchical condition clustering (HCC) indicated a primary separation of samples based on developmental stage. Intriguingly, a further separation was observed within the adult samples. This separated the samples into two groups that accorded with the time of year the adults were harvested from culture for experimental use in the laboratory experiment (Figure 4). Earthworms for two experiments (with Cd and FLA) were harvested in November and those for the third (with ATZ) were harvested in late December. Transcript profiling may therefore also be able to provide molecular signatures relating to seasonally-responsive biology and behaviour.

Substantive differences were evident between the juvenile and adult groups, with ~45% of the reporters that consistently generated features passing automated and manual quality criteria yielding measurements showing >1.4-fold expression change between the two conditions. A t-test identified 747 genes with significantly altered transcript levels (p < 0.01 following Bonferroni multiple sample correction; a full list is provided in Additional File 5). Using only these significantly changed genes, HCC separated all samples by developmental stage (Figure 4). Annotation of genes with changed expression levels between juveniles and adults indicated that transcripts associated with macromolecular biosynthesis, energy production, and connective tissue synthesis (all processes associated with rapid growth rate) were over-expressed in juveniles (Additional file 6). Also over-expressed in juveniles was an invertebrate oxygen-carrier, erythrocruorin, which showed up to 5-fold bias. Genes with higher expression in adults reflected activities associated with turnover of cellular components, including biopolymer metabolism, catabolism, and hydrolase activities. Multiple isoforms of ferritin heavy chain (FTH1) were over-expressed 50- to 100-fold in adults. Although a library was constructed from the anterior segments (segments 1–33) to sample from reproductive tissues (clitellum, spermathecae and ovaries; Table 1: Library 4) we were not able to identify many transcripts associated with the biological process of sexual reproduction. A notable and intriguing exception is an homologue of the estrogen receptor co-activator (LRC06848) which, although originally identified from the juvenile library (Table 1: Library 3), did show a mean >3-fold, and statistically significant, increase in expression level in the adult samples.

While assessment of GOSlim annotations of individual clusters by library yielded similar patterns of inference as to biology (see Figure 2), the granularity of the EST dataset made it a far less sensitive probe of animal physiology than the microarray analyses. Importantly, the microarrays permitted assessment of expression of genes sampled only a few times in the EST strategy.

### Transcript responses of *L. rubellus *to xenobiotic exposure

**Figure 5:**
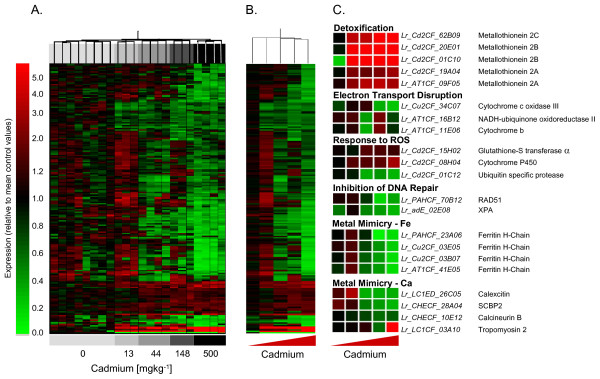
**Transcriptional responses of adult *L. rubellus *exposed to a series of concentrations of Cadmium**. The array data used for this analysis were normalised per chip and per gene median polishing, expressed relative to the control samples; data from poor quantity spots were removed, and genes showing significant (p < 0.05) differential expression in response to Cadmium identified using ANOVA analysis with Benjamini and Hochberg False Discovery. Hierarchical clustering was performed using a distance algorithm for both conditions and genes. Panel A shows hierarchical clustering of the individual samples, whilst Panel B provides that hierarchical clustering of average expression generated by treatments. Panel C displays the average expression of a series of important functional groups pertinent to Cadmium toxicosis. Full details of the provenance of these reporters are provided in Additional File 10.

**Figure 6:**
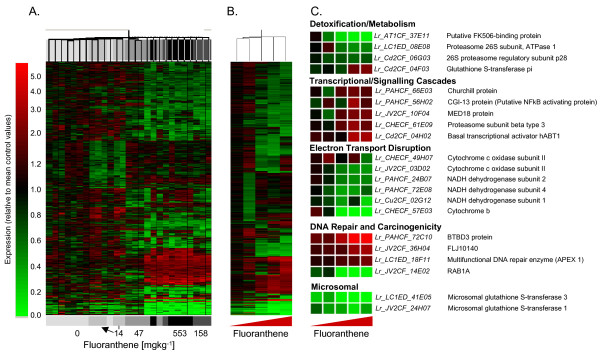
**Transcriptional responses of adult *L. rubellus *exposed to a series of concentrations of Fluoranthene**. The array data used for this analysis were normalised per chip and per gene median polishing, expressed relative to the control samples; data from poor quantity spots were removed, and genes showing significant (p < 0.05) differential expression in response to Fluoranthene identified using ANOVA analysis with Benjamini and Hochberg False Discovery. Hierarchical clustering was performed using a distance algorithm for both conditions and genes. Panel A shows hierarchical clustering of the individual samples whilst Panel B provides that hierarchical clustering of average expression generated by treatments. Panel C displays the average expression of a series of important functional groups pertinent to Fluoranthene toxicosis. Full details of the provenance of these reporters are provided in Additional File 11.

**Figure 7:**
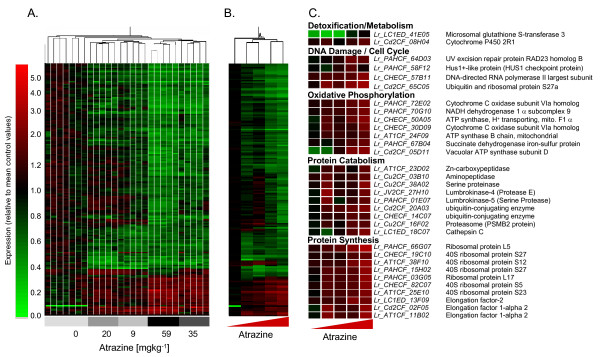
**Transcriptional responses of adult *L. rubellus *exposed to a series of concentrations of Atrazine**. The array data used for this analysis were normalised per chip and per gene median polishing, expressed relative to the control samples; data from poor quantity spots removed and genes showing significant (p < 0.05) differential expression in response to Atrazine identified using ANOVA analysis with Bonferroni False Discovery. Hierarchical clustering was performed using a distance Algorithm for both conditions and genes. Panel A shows hierarchical clustering of the individual samples, whilst Panel B provides that hierarchical clustering of average expression generated by treatments. Panel C displays the average expression of a series of important functional groups pertinent to Atrazine toxicosis. Full details of the provenance of these reporters are provided in Additional File 12.

Dose-response transcription profiles were determined for three xenobiotics from different chemical classes: inorganic (cadmium), organic (fluoranthene), and agrochemical (atrazine) (details of experimental conditions and design are available in ArrayExpress accessions E-MAXD-34, -31 & -36 respectively). Those data passing quality control criteria were analysed as independent compound exposures employing a standard normalisation, followed by filtering to select those reporters that passed automated and manual quality criteria and showed a >1.4-fold expression change in all conditions. This dataset was scrutinised further to identify statistically significant expression changes. Gene lists for each compound exposure are given in Additional Files 7, 8 and 9. The resultant transcript profiles for replicate samples for each exposure concentration for each compound were hierarchically clustered (Figures 5, 6, 7, Panels A), the average transcript profiles computed for each exposure condition (Figures 5, 6, 7, Panels B), and key genes providing functional insights into the response profile identified (Figures 5, 6, 7, Panels C) (see Additional files 10, 11 &12).

For each compound, samples from the same exposure condition were more closely related to each other than to other doses (Figures 5, 6, 7, Panels A). The only exception was seen in FLA, where the groups exposed to the two highest concentrations (158 and 533 ppm) are inter-mixed, but clustered separately from the remaining exposure conditions (Figure 6, Panel A). For all compounds, HCC analysis indicated a second tier of clustering, separating controls from the two lower doses and both of these groups from all replicates for the two higher doses. Since all exposure experiments were designed with a logarithmic dose series across the full sub-lethal exposure range (see Figure 3), this similarity in clustering pattern may reflect similar patterns in transcript change associated with the extent of toxic effects.

### Molecular insights into xenobiotic response pathways

**Figure 8:**
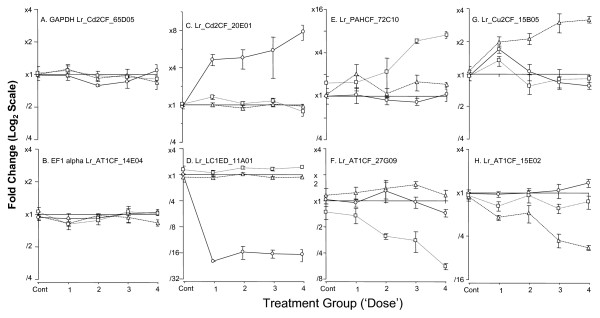
**Transcript profiles for individual gene responses to xenobiotic exposure**. The expression profiles of eight specific reporters are provided to illustrate changes in expression during xenobiotic exposure relative to that observed in control organisms. Transcript changes are shown in response to; Cadmium (exposures 0, 13, 44, 148 and 500 mg/kg) represented by open circles connected by solid lines; Fluoranthene (exposures 0, 14, 47, 158 and 553 mg/kg) designated by open squares and doted line and Atrazine (exposures 0, 9, 20, 35 and 59 mg/kg) represented by open triangles connected by dashed lines. Panels A & B show the profiles of two invariant control genes [73] GAPHD (Lr_Cd2CF_65D0; GenBank accession: DR077591, Cluster:LRC04105_1) and EF1α (Lr_AT1CF_14E04; GenBank: CO047675, Cluster: LRC04881_1). Panel C and D depict the expression profiles for Metallothionein 2B (Lr_Cd2CF_20E01; GenBank: CF611058 Cluster:LRC01607_2) and Laminin (Lr_LC1ED_11A01 GenBank: CF416412 Cluster:LRC01132_1), genes that exhibit compound-specific up- and down-regulation, respectively, in response to cadmium. Panels E & G show reporters Lr_PAHCF_72C10 (GenBank: CO048307, Cluster:LRC05297_1) and Lr_Cu2CF_15B05 (GenBank: DR008743, Cluster:LRC08839_1) specifically induced by Fluoranthene and Atrazine, respectively. Panels F & H show reporters Lr_AT1CF_27G09 (GenBank: CO046812, Cluster:LRC04302_1) and Lr_AT1CF_15E02 (GenBank: CO047812, Cluster:LRC04973_1) exhibiting reciprocal repression by exposure to the PAH and herbicide, respectively.

In our study, earthworms were maintained in the presence of each chemical for a relatively long period (28 days) compared to more standard, acute assays (usually 48 hours on filter paper or 14 days in soil). This extended exposure was intended to permit the measurement of transcriptome responses at a physiological acclimated plateau rather than in a dynamic flux state associated with the initial exposure stress. We have mined these transcriptional changes to provide insights, some established and some novel, into the mode of action of each xenobiotic. Since our array only reports on a subset of the complete *L. rubellus *transcriptome, and does not address alternative splicing or posttranscriptional modifications, the insights derived are by default fragmented. Inevitably, our analysis exploits primarily a small proportion of well annotated genes, including invariant controls (Figure 8, Panels A and B) and compound responsive transcripts (Figure 8, Panels C and D). However, the majority of genes that show statistically significant relationships between xenobiotic dose and transcript levels do not have informative similarity to known proteins (Figure 8, Panels E and H). While we can annotate these genes as 'regulated by xenobiotic exposure', we are obliged to leave assignment of their molecular functions and relationships as key avenues for future work on the molecular physiology of *L. rubellus*.

### Cadmium: a response to inorganic exposure

Cadmium is a recognised carcinogen with a well described underlying molecular aetiology (for reviews see [25-27]). It has been shown to induce oxidative damage, modulate DNA repair and interfere with metabolism of essential metal ions, including Fe, Ca, and Zn, in yeast and cultured cells. For *L. rubellus *the annotated cDNA microarray identified dose dependent transcript profiles congruent with these molecular mechanisms, together with tantalising novel insights into the secondary impacts of chronic Cd exposure on the earthworm.

**Figure 9:**
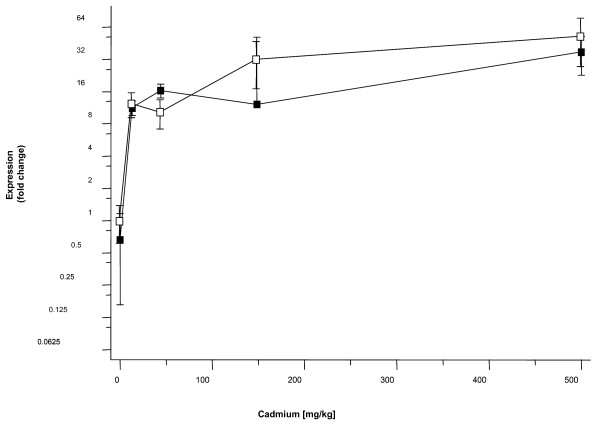
**Relative correlation between microarray and qPCR data for Metallothionein*-2 *expression in *L. rubellus***. The relative expression profiles for Metallothionein*-2 *following sublethal cadmium exposures measured either using microarray analysis (solid squares) or with real-time qPCR analysis (open squares, data expressed relative *β*-actin). Data points represent mean fold change values ± SE.

The cluster of transcripts exhibiting the most significant dose dependent induction in response to cadmium were members of the key protective and detoxification pathway for Cd, namely, isoforms of the small, cysteine-rich, metal-binding protein, metallothionein (Figure 5, Panel C). The role of transcriptional up-regulation of metallothionein isoforms in cellular detoxification of cadmium in earthworms has been well characterised [9,13,15]. The induction profile was confirmed by QPCR analysis on independently transcribed samples of the total RNA used for microarray analysis (Figure 9). The highly significant correlation (R = 0.965 p < 0.005) of the microarray and QPCR data within the experiment, and the agreement with the previous independent studies cited above, provides confirmation of the validity of our microarray findings.

Cd also inhibits the electron transport chain, and causes the generation of reactive oxygen species (ROS). It has been proposed that the specific sites of impact of Cd are in complexes II (ubiquinone oxidoreductase) and III (cytochrome c), with the majority of ROS production being associated with the inhibition of complex III [28]. The array results supported this model, showing a significant dose-dependent reduction of a cytochrome c oxidase from complex III, and a modulation of the expression of NADH-ubiquinone oxidoreductase II and cytochrome b (although the latter effects are dose specific) (Figure 5, Panel C). The observed inductions of a cytochrome P450 and glutathione-S transferase (GST) alpha by Cd (Figure 5, Panel C) are perhaps a response to ROS-induced damage, and have previously been observed in yeast and rat liver, respectively [29,30]. Intriguingly, the induction of the cellular response cascade to Cd-induced oxidative damage has been linked to increased levels of the transcription factor Nrf2 through direct Cd interference with ubiquitin-mediated proteolysis [31]. The earthworm array indicates a transcriptional down-regulation of an ubiquitin specific protease (Figure 5, Panel C), and it will be important to establish whether this inhibition is linked to modulation of Nrf2 degradation.

Although at high concentrations Cd may impact on DNA directly, causing DNA conformational alterations, at lower levels it causes cytotoxic effects through inhibition of DNA repair [32]. Cd affects both early and late steps of nucleotide excision repair, and also inhibits repair of DNA strand breaks and post-replication mismatch repair. However, the exact mechanisms underlying these effects have not been described. Our data may provide a novel insight into this process, as *L. rubellus *genes with significant similarity to RAD51 [33] and XPA [34], key components of repair pathways for double stranded DNA breaks and excision repair respectively, are down-regulated in response to Cd (Figure 5, Panel C). Since both RAD51 and XPA associate with Zn it is conceivable that Cd may disrupt or inhibit these key components of the DNA repair pathway through molecular mimicry. It will be important to confirm these observations within other species.

As established in model species, molecular mimicry arguably provides the most wide-ranging vehicle through which Cd can elicit toxic effects on a biological system. Ca, Zn and Fe are all employed as key cofactors in proteins that function as structural components, second messengers, transcriptional regulators and redox centres. The binding of Cd to these metallo-proteins will have wide-ranging effects. The established link between Cd toxicity and disruption of Fe metabolism leading to anaemia was reflected within the array data through the significant down-regulation of heavy chain ferritin (Figure 5, Panel C). Whether this is a direct effect of Cd or a result of disruption of general Fe homeostatic sensing (through the known interaction of Cd with the iron regulatory protein [35]) is unclear. The impact of Cd on Ca metabolism was evident on the arrays as a negative transcriptional influence on the Ca-binding proteins calcineurin, calexcitin and sarcoplasmic Ca-binding protein (SCBP2) (Figure 5, Panel C). Furthermore, the long-term effects of Cd on the cytoskeleton [36] were also reflected in the Cd-specific up-regulation of tropomyosin 2 (Figure 5, Panel C).

### Fluoranthene: a representative polycyclic aromatic hydrocarbon toxin

PAHs are lipophilic and so can incorporate into and disrupt the functions of biological membranes [37]. Low-level exposure to PAH has been linked to several adverse effects, including carcinogenesis [38], teratogenesis [39], and induction of cardiovascular disease [40,41]. In mammals the molecular trigger coordinating cellular responses to certain PAHs is the upregulation of aryl hydrocarbon receptor (AhR) activity. This activation is not mediated transcriptionally, but results from reduction of the proteolytic turnover of AhR by the ubiquitin-26S proteasome pathway [42]. In *L. rubellus*, exposure to our model PAH, FLA, caused the down-regulation of two members of this proteolytic pathway, proteasome 26S sub-unit ATPase 1 and 26S proteasome regulatory sub-unit P28 (Figure 6, Panel C). A second regulatory mechanism is moderation of an AhR – heat shock protein 90 complex by FK506-binding proteins (FKBP) [42]. In *L. rubellus*, FLA exposure caused a >30-fold down-regulation of one FKBP isoform (Figure 6, Panel C). These observations suggest that *L. rubellus *orchestrates its response to FLA through these established mechanisms.

AhR is a transcriptional regulator. Specific inhibition of degradation increases the cellular concentration of AhR, and thus causes transcriptional up-regulation of a set of target metabolic and detoxification mechanisms. Classically these responses have been associated by the induction of cytochrome P450 reductases, but recently alternative metabolic pathways have been identified, including GSTs and NAD(P)H:quinone oxidoreductases [43]. In *L. rubellus *exposed to FLA, this detoxification response is exemplified by the up-regulation of a homologue to a Pi class GST, a cytosolic isoform that has previously been linked to PAH metabolism [44] (Figure 6, Panel C). AhR independent pathways activated by PAH include those involving early growth response factor 1 and peroxisome proliferation-activated receptor alpha [45,46].

Biotransformation of PAH leads to the generation of reactive oxygenated metabolites (ROM) and ROM-mediated oxidative stress [43]. This process activates a broad spectrum of pathways associated with inflammation and hypoxia. This is reflected through the up-regulation of factors involved in the positive regulation of global transcription, including homologues of hABT1, Churchill protein and CGI-13 (Figure 6, Panel C). A specific link has been established between the activation of hypoxia-inducible erythropoietin and the activation pathways under the control of NFkB [42,47]. NFkB serves as a second messenger to induce a series of cellular cytokines in the response to cellular damage invoked by, amongst others, reactive oxygen species generated by PAH exposure. The observed up-regulation of a CGI-13 protein homolog, a putative NFkB activating protein, in response to FLA exposure supports the assertion that the earthworm is responding to ROM-induced stress (Figure 6, Panel C).

The direct cellular impacts of PAHs have been associated with: (1) mitochondrial dysfunction or decay leading to the uncoupling of mitochondrial respiration, and inhibiting electron transport; (2) direct genotoxic damage through DNA adduct formation; and, (3) hypoxia and ROS and ROM generation [42,43]. All three processes are clearly evident in the earthworm transcript response profiles. The mitochondrial-encoded genes NADH dehydrogenase subunits 1,2 & 4, cytochrome b, and cytochrome c oxidase subunit II, showed significant down-regulation, with those involved in DNA damage repair, such as BTBD3 protein and APEX nuclease (multifunctional DNA repair enzyme) 1, were significantly up-regulated (Figure 6, Panel C).

Overwhelmingly, the responses observed within the earthworm are comparable with studies performed in mammalian systems. However, one area where these annelids show a distinct difference is in the down-regulation of microsomal-based metabolic machinery, including phase I (cytochrome P450 mixed function oxygenase (MFO) enzymes) and phase II (including GST) detoxification reactions. Significantly, homologues of two microsomal GSTs show significant down-regulation (Figure 6, Panel C). Furthermore, no homologue of cytochrome P450 isoform 1A (CYP1A) has so far been identified in *L. rubellus*. This finding is supported by a number of studies in earthworms [48,49] which did not observe an increase in enzyme activity using assays which target microsomal cytochrome P450 enzymes involved in biotransformation of organic xenobiotics (CYP1A activity, measured using the EROD assay). However, other studies have shown induction of CYP1A (through measuring CYP1A immunopositive protein) in invertebrates, such as molluscs, following exposure to organic chemicals [50,51]. While the role of the cytochrome P450 MFO system is well understood in vertebrates, the data presented here suggest that there is a subtly distinct, hitherto undescribed variant mechanism active in *L. rubellus*.

### Atrazine: an S-triazine-ring herbicide

Compared to the significant body of research relating to the gross toxicology of ATZ (see PAN Pesticides Database [52] for literature review of toxicity and ecotoxicity data), very little is known of the molecular pathways underlying these physiological effects. It has been proposed that ATZ breakdown is linked to standard organic phase I and phase II metabolic pathways, implying that cytochrome P450 reductases and GST may be invoked as the primary detoxification mechanisms. This proposition is supported by the *L. rubellus *data, which indicated transcriptional up-regulation of representative P450 and GST isoforms (Figure 7, Panel C).

ATZ is classed as a potential carcinogen with teratogenic activity [53]. On exposure to ATZ, *L. rubellus *showed up-regulation of a number of genes that are associated with DNA damage, such as UV excision repair protein RAD23 homologue B and HUS1 like protein (HUS1 checkpoint protein). Furthermore, enzymes directly involved specifically in UV-responsive excision repair are also up-regulated, along with genes involved with chromatin remodelling (Figure 7, Panel C). An impact on the transcript levels of genes involved in controlling the cell cycle was also observed. This may indicate a link to mechanisms that control cell cycle progression in response to genetic integrity of the cell (Figure 7, Panel C).

As an herbicide, the mode of action of ATZ is the disruption of electron transport by targeting chloroplast photosystem II. Inhibition occurs at the level of protein-bound plastoquinone B [54]. The response of *L. rubellus *to ATZ measured on the arrays indicated a significant up-regulation of several members of the oxidative phosphorylation pathway and of the tricarboxylic acid cycle (Figure 7, Panel C). This may indicate that, at high doses, ATZ elevates mitochondrial electron transport and alternative routes of ATP generation to compensate for a partial uncoupling of oxidative phosphorylation.

However, in the *L. rubellus *response to ATZ, the largest and most significantly overrepresented group of genes were associated with protein synthesis and catabolism (Figure 7, Panel C). The up-regulation by ATZ of the ubiquitin-conjugating enzyme E2 which is central for the targeting of protein for degradation in concert with ubiquitin-protein ligases (E3s) via the HECT pathway is indicative of this raised catabolic state [55]. This was counterbalanced by observation of increases in a large number of genes associated with up-regulated protein synthesis such as ribosomal proteins and amino acid transporters. A possible explanation for these observations might be an increase in the generation of incorrectly folded proteins. Degradation of these non-functional proteins and their re-synthesis would account for the transcript changes observed.

## Conclusion

A thirty-fold accretion in the genetic knowledge-base for any species belonging to an acknowledged keystone taxon that has been previously neglected in sequencing studies might reasonably be expected to yield general and specific insights into individual species and group systematics. Indeed, a recent phylogenetic revision of the animal kingdom, partly based on the *L. rubellus *ESTs, defining 8,129 gene objects generated as a component of this work, clearly grouped earthworms with flatworms and molluscs in a superphylum, the Lophotrochozoa, alongside the superphylum Ecdysozoa, to form one of the two major animal divisions, the Protostomia [56,57]. The sequencing work described here has, thus, already made a valued contribution to understanding the evolutionary relationships between animal phyla.

The availability and functional annotation of the EST resource, and the subsequent clustering to identify a set of gene objects has allowed the first high density microarray for *L. rubellus *to be fabricated. The development of this 8,000-feature array provides a valuable resource to the earthworm community for future studies in areas such as tissue regeneration and immune system function, for which earthworms are already in use as model systems [58,59]. Through experimental studies, we have clearly demonstrated the capacity of the microarray to provide novel insights by investigating the molecular basis of the responses of the species to environmental perturbations. All our data and analyses have been collated and presented on a web-available database, LumbriBASE [19], that permits querying of sequence, annotation and microarray data through an easy-to navigate interface. These analyses promise radical changes in the use of earthworms for the biological assessment of soil contamination. Thus, not only have we revealed differences in the qualitative and quantitative expression patterns of a high proportion (~40 to 50%) of all *L. rubellus *genes following exposure to three chemicals of contrasting modes of action, but it has also provided novel molecular insights that complement the outcomes of previous, targeted functional biochemical and metabolomics studies on this species [5,13]. With this expanding set of tools now available, it can only be assumed that *L. rubellus *will increasingly be viewed as a powerful tool through which to probe, with an evolutionary perspective, the biochemical, physiological and evolutionary responses of a 'soil engineering' species to environmental perturbations and changes.

## Methods

### *L. rubellus *sourcing, culturing and xenobiotic exposure

*L. rubellus *used for library construction and all exposure work were acquired from an uncontaminated field site by a commercial supplier (Neptune Ecology, Ipswich, UK), with the exception of the worms used for constructing the Cd library which were collected from a control field site at Dinas Powys, South Wales (Ordnance Survey Grid Reference ST146723). After collection, all worms were kept on a 1:1:1 mix of loam soil:peat:composted bark for a minimum of 4 weeks. Two weeks before the start of each experiment, all required worms were transferred to a standard loam soil (pH 7.1, 5% organic matter) (Broughton Loams, Kettering, UK) with 3% composted bark (LBS Horticultural, Colne, UK) added and maintained at 15–20°C under a 16:8 light:dark regime to allow them to acclimatise to the test soil and conditions.

Worms used for generation of the control library and body section libraries were selected following acclimatisation without further treatment. These were keyed out to confirm species identification and a set of healthy individuals dissected if required and then processed for total RNA extractions. Breeding pairs of adults were assembled and placed on clean soil and checked every four weeks and laid cocoons collected. A random selection of these cocoons were incubated for a further five weeks before being dissected and the embryonic tissue collected for use in constructing the late cocoon library. The remaining cocoons were hatched and the worms grown for ~40 days to yield a juvenile cohort for library construction. Libraries made using exposed worm tissue were made from pre-acclimatised adults kept in cadmium (Cd; 50, 200 and 600 ppm), fluoranthene (FLA; 62, 140, 316, 711 and 1066 ppm), atrazine (ATZ; 12 and 35 ppm) and copper (Cu; 40, 160, 460 and 480 ppm) spiked, bark-amended loam soil for 28 days.

The same amended loam soil was used to generate sample for all microarray experiments. Juvenile samples were obtained from worms grown individually from hatching [60]. These were monitored until each reached approximately 300 mg weight at which time they were snap frozen at a designated diurnal time and then processed for microarray analysis.

Adult worms were exposed to cadmium, fluoranthene and atrazine in separate exposures designed from published and range-finder test data to represent a series of soil concentrations ranging from unexposed (controls) to just below the lethal level. The exposure concentrations (in mg/kg soil) used were:

Cd: 0, 13, 43, 148, 500

FA: 0, 13.8, 46, 158, 533

AZ: 0, 9.4, 20.7, 35, 59.

Eight replicate of controls and five replicates of spiked treatments were used for each experiment. Cd was spiked into soil in the water that was required to raise the soil moisture content to the required level (33% wet weight = approximately 60% of water holding capacity), fluoranthene and atrazine were spiked as solutions in acetone and ethanol, respectively. Each soil, including treatment controls, was amended with the required amount of "make-up" solvent to ensure that all soils received the same volume of solvent as the highest concentration. After dosing, all soils were vented for at 72 hours to allow evaporation of the carrier. Soil were then wetted to the same moisture level used in the Cd test.

The adult test was conducted according to a 28 day protocol [60] using the design outlined in Figure 3. For logistic reasons (principally the need for time-synchronised sampling), the experiments for each compound were run in series rather than parallel, and thus the tests were conducted at different times of the year. Exposures of batches of 8 worms per treatment replicate were conducted for 28 days at 15°C under a 16 hr light: 8 hr dark regime. Each batch of worms was fed at 0 and 14 days with 5 g (dry weight) of horse manure spiked to the same concentration as the test soil. After 28 days, at an identical designated diurnal time (midday ± 30 min), worms were retrieved from the soil, immediately snap frozen in liquid nitrogen, and stored at -80°C. During processing each worm was visually inspected for phenotypic characters (presence of skin lesions, scarring of the clitellum, presence of body constrictions, loss of torpor, and reduced vigour) and given a "condition index" score ranging from 1 (pristine) to 5 (very poor for many characters). Percent survival of the experimental cohort was recorded and the soils sieved to collect cocoons in order to determine reproductive rate. For each experimental replicate, three worms were pooled to give a single biological sample for array analysis. The worms chosen were the three that had the highest condition score at the end of the test. Although this selection may have favoured the inclusion of more tolerant genotypes, it avoided the inclusion of worms that had lost condition for non-treatment reasons (such as disease or parasitism) during exposure.

### Total RNA extractions

All fresh tissue was immediately homogenized in Tri-reagent (Sigma-Aldrich) and stored at -80°C before processing. Stored tissue was crushed to fine powder under liquid nitrogen and homogenised in Tri-reagent at 50 mg per ml (Sigma Chemicals, Poole, UK) for 2 minutes at high speed using an Ultra-Turret^® ^T18 homogeniser (IKA, Stauffer, Germany). Total RNA was extracted using standard protocols [61,62] followed by additional purification with RNAeasy kits (Qiagen), and quantification by spectrophotometry.

### Library construction and screening

Nine cDNA libraries were constructed from control (unexposed) and exposed animals (Table 1 and Additional File 1). Library 1 was constructed from healthy control adults. Library 2 was constructed from embryonic material collected from pair breed cocoons (see above), and Library 3 from juveniles, each obtained from hatchlings reared from laid cocoons. A separate cDNA library (Library 4) was constructed from the anterior (pre-clitellum) portion of healthy adults. Representative, healthy individuals from each concentration of each xenobiotic exposure were processed to generate total RNA. Xenobiotic compound-specific RNA pools were then constructed by combining an equal mass of RNA from each dose. These pools were then used to construct additional cDNA libraries: Cd Library 5, FA Library 6, AZ Library 7 and Cu Library 8. mRNA for these libraries was purified using oligo d(T) cellulose columns (Amersham Life Sciences). Libraries in the plasmid vector pBluescript II (Libraries 2–8), were generated using the pBluescript II XR cDNA library kit (Stratagene). Library 1 was generated in the phagemid pBluescript II SK+ (Stratagene), and subsequently recovered as plasmid (pBluescript II SK+) by mass excision. A suppression subtractive hybridisation library (Library 9) was also generated (using the Clontech PCR-Select cDNA subtraction method) to enrich for genes involved in reproduction with driver cDNA generated from posterior segments and tester cDNA from the segments containing the clitellum and seminal vesicles. The population of amplified enriched fragments was ligated into pGEM-T (Promega).

### EST sequencing

Plasmid clones were picked, cultured and cDNA inserts amplified by PCR using vector primers in a final volume of 100 μl following standard methods [63]. Products were purified, and the sample concentrated to 20 μl, using Montage Multiscreen PCR Cleanup Plates (Millipore). The PCR products were sequenced from the 5' end of the cDNA insert using a T7 primer and BigDye and DyenamicET sequencing reagents, and analysed on an ABI 3730 sequencer by the Edinburgh School of Biological Sciences Sequencing Service.

To improve the yield of novel sequences, a screen was introduced to remove unsuccessful insert cDNA amplifications and highly abundant transcripts. The success of each amplification was first verified by visualisation on 96 well 2% E-gels (Invitrogen) and subsequently an aliquot of each amplicon was printed on an array and hybridised (as described below) to a fluorescently labelled mixture representing the 50 most abundant transcripts identified in the first 3966 *L. rubellus *ESTs. The longest EST representative of the 50 clusters containing the greatest number of ESTs was selected and insert cDNAs labelled in 10 batches. For each mix of 5 probes, 0.2 μl of a 1/100 dilution of each probe insert was PCR amplified using primers designed to the vector/adaptor interface. In this labelling PCR mix, added dTTP was reduced to 0.3 nmoles and 1 μl of Cy5-dUTP (Amersham) was added. Unincorporated dye was removed using GFX columns (Amersham) and the products eluted in 60 μl. A mixture (1 μl of each batch) of the abundant transcript probes was hybridised to the arrayed PCR products in the presence of the amplification primers to reduce cross hybridisation. cDNA inserts that had good amplification but did not hybridise to the abundant transcript probes were picked robotically to new microtitre plates (Multiprobe II HT EX liquid handling system, Parkard) and processed for sequencing (see above). The 100 μl volume PCR amplifications yielded sufficient product for quality assurance and sequencing (~15 μl) and a large stock for subsequent microarray fabrication.

### EST processing and annotation

Primary chromatograms were processed using trace2dbEST, a perl pipeline script that uses Phil Green's phred and user-supplied cut-off information to base call and trim sequences, formatting them for submission to GenBank dbEST. All 17,225 ESTs have been submitted. The ESTs were then clustered and annotated using PartiGene [17]. PartiGene first groups ESTs into clusters that putatively derive from one transcript using CLOBB [64], and then derives a consensus sequence for each cluster with Phil Green's phrap. The EST translation tool prot4EST [18] was used to predict putative protein translations for each consensus sequence. Each consensus was compared to the UniProt protein database [65], and to custom databases of other annelid and model organism sequences, using BLAST [66]. Further annotations were derived using GOtcha, which provides gene ontology annotation with robust quality scoring [21], and modules in the annot8r series (Ralf Schmid and M. Blaxter, in press) that provide annotations referring to protein domains and families (via Pfam and Interpro) [67], enzyme commission (EC) identifiers, the KEGG metabolic pathways database, putative cellular location (via PSort and SigP) [68,69], physical properties (via ExPasy) [70], and secondary structural predictions (using e.g. TmHMM).

### A unified database: LumbriBASE

All the sequence data and associated annotations were collated in a PostgreSQL relational database called LumbriBASE [19]. The database was also populated with post-primary analysis microarray expression data (see below), and was used in intensive data exploration. Public access to the database is provided through a scripted internet-accesible interface using PHP, CGI and java scripts. LumbriBASE also houses analyses of EST data from other annelids derived from submissions to GenBank dbEST: the earthworm *Eisenia andrei *(1108 ESTs), the polychaete *Nereis virens *(6978) (Olive and Blaxter, unpublished data), and the leech *Haementeria depressa *(891 ESTs).

### Microarray experimental design

A reference design was employed for profiling *L. rubellus *transcript expression levels. The reference sample consisted of 65–70 base-long oligonucleotides designed against vector sequences found between the amplification primer and the inserted cDNA. The sequences of these primers are given in Additional File 2. An equimolar quantity of two oligonucleotides was used in order to represent the three different vectors exploited for library construction. Use of this reference meant that essentially all reporter spots could be called as positively hybridising in the reference, extending the ability of the array to report on even low hybridisation signals in the experimental samples. In addition, as this reference could be easily chemically synthesised and therefore replicated, this design permitted comparison of all samples against a common reference within the present experiment, and can be extended for future work. The hybridisation probe included ~30 pmol of Cy3 labelled target RNA and 1 pmol of each oligonucleotide.

### Microarray fabrication

A representative EST (usually the one inferred to be the longest) was selected from each of the 8,029 clusters assembled from EST sequence data. Aliquots (5 μl) of the amplified and concentrated products were transferred to 384 well plates and mixed with an equal volume of DMSO. These composite plates were then used to print onto Ultra-GAP glass slides (Corning) using 48 SMP3 pins (Telecham) mounted in a Spotarray 72 (Perkin-Elmer). This printing regime yield spots of approximately 120 μm in diameter. Landmarks were introduced at the left had corner of each sub-array (and thus evenly spaced across the whole array) by the introduction of 5 replicates of the Lucida Scorecard (Amersham). The Lucida Scorecard is a selection of heterologous gene reporters which show no cross reactivity to earthworm transcripts (data not shown). Reporters were cross-linked to the surface by baking at 80°C for 2 hours, and UV cross linking. Slides were stored in the dark and under a vacuum until required.

### Microarray probe preparation

The Lucida Scorecard test spike (Amersham Life Sciences) was added to 10 μg of total sample RNA prior to oligo-d(T) reverse transcription and coupling to Cy3 using an indirect amino amyl procedure [71]. Labelled targets were separated from unincorporated dye by precipitation and separation on a GFX column (Amersham Life Sciences). Yields of cDNA and incorporated dye were calculated by measuring absorption at 260 and 550 nM. The quality of the labelled targets was assessed subjectively by separation on a 2% agarose gel poured on a microscope slide and visualised using a LSIV laser scanner. Only labels that demonstrated fluorescent incorporation in a wide size range of cDNAs and where incorporation efficacy exceeded 20 pmole CyDye/μg cDNA were used for hybridisation [71].

### Microarray hybridisation and quality control

Slides were pre-treated by immersion at 42°C in block buffer (5 × SSC, 0.1% SDS and 1% BSA) for 45 min. Slides were then washed in 0.2 μm filter-sterilised water and dried using compressed air. Labelled target (representing 30 pmole of Cy3) was mixed with the common reference (representing 30 pmole of Cy5) and 0.1 nmoles of oligo d(T)_17 _before denaturation at 95°C for 3 min. This was immediately introduced onto the slide surface in the presence of 50% formamide, 10 × SSC and 0.2% SDS in a total volume of 40 μl. A second slide was applied to the surface of the array in such a way as to exclude air bubbles and the hybridisation completed in a humidity chamber at 42°C overnight (18 hr). Slides were separated in 1 × SSC, 0.2% SDS at room temperature and washed in the same buffer for 10 min at 55°C. They were subsequently washed twice in 0.1 × SSC, 1% SDS at 55°C prior to a final room-temperature rinse in 0.1 × SSC. Slides were dried with compressed air and an array image acquired using a ScanArray Express (Perkin-Elmer).

Analysis of calibrators exploited 10 "alien" RNA spikes (components of the Amersham Lucidea Scorecard) introduced at known concentrations, between 1 pmole and 30 nmole, prior to labelling and each hybridizing to 10 replicate reporter spots on the array. Image analysis of the signals generated by these reporters was performed for each array to determine the sensitivity and relationship between RNA concentration and fluorescent signal. Hybridisations showing non-linear response, or where the detection limit was below the 10 pmole Lucidea ScoreCard calibrator, were removed from subsequent analysis (For representative calibrator analysis, see Additional File 3).

### Statistical analysis of microarray data

Array images were subjectively quality controlled for artefacts that would compromise quantification such as background effects and spot morphology. Subsequently, the calibration standards from the Lucida Scorecard were analysed to objectively assess the sensitivity range and to define both saturation and background readings. The images were analysed using Imagene (Biodiscovery), using the default flagging and segmentation settings, and subsequently checked by eye. All subsequent analysis was performed in GeneSpring 7.3 (Agilent Technologies, Palo Alto, CA). Array data passing these quality standards were processed by background subtraction and generation of Cy3/Cy5 ratio. Data was then normalised within an experiment using median polishing (per gene and per chip) normalization implemented using the default method within GeneSpring Software (Agilent Technologies, Palo Alto, CA) incorporating only data where Imagene had flagged the spot good (0 flag) and the background-subtracted data exceeded 100 relative light units. Where appropriate, data was also normalised by dividing by data derived from the relevant biological controls. The processed data distributions were visualised using a box plot to establish whether each chip's data distribution exhibited comparable median and quartile ranges, and data not complying was further reviewed for the quality for the raw data. Any data compromised by experimental artefacts was removed from the analysis (Box plots for each experiment are given in Additional File 4).

All statistical analyses within an experiment were performed on subsets of genes where the data was flagged as good in a number of arrays representing at least the minimum biological replicate size. In this way, poor data was removed without thereby removing genes that were only significantly expressed in one experimental condition. Further filtering was performed prior to statistical analysis to remove genes that displayed less than a 1.4 fold change to any condition within the experiment to minimise false discovery. Statistical tests used and their parameters are presented with the relevant data. Annotation used for analysis was generated from LumbriBASE (see above). Relevant abstractions of the annotation is provided for all genes exhibiting significant transcript changes between given biological conditions within Additional Files 5 and 6. These tables list most the significant homologue identified within the set of human proteins (from SwissProt), the global protein database (UniProt) and the global nucleotide database (EMBL) using BLASTX or BLASTN, as appropriate, with an E-value cut-off for BLASTX of 1e-05, and of 1e-65 for BLASTN. A brief description of the match, its accession number and the BLAST score and E-value are provided. Ontological bias analysis performed when analysing the developmental transcript changes exploited the SwissProt accession numbers of those genes assigned a human homologue genes within the Database for Annotation, Visualization and Integrated Discovery (DAVID) environment [72].

### PCR validation of individual gene expression profiles

Validation of array results focused on a confirmation of expression patterns by real time quantitative reverse transcriptase PCR (Q-RT-PCR). Analysis by Q-RT-PCR was conducted for two well characterised earthworms gene, metallothionein-2 and β-actin, for which Q-RT-PCR primers were developed and fully validated in previous work [62]. All quantifications were conducted using a cDNA template generated from the sample extract of total RNA used for the microarray hybridisation. Reverse transcription of a 2 μg sample of collected total RNA was conducted at 42°C, anchored by oligo (dT_17_) primer and random hexamers (N_6_) and using Moloney-Murine Leukaemia Virus reverse transcriptase for second strand synthesis.

Q-RT-PCR quantification were conducted using custom designed primers and the universal SYBR^® ^Green Supermix (BIO-RAD, UK). All reactions were optimised to yield an exponential amplification of the target gene. Q-RT-PCR quantification was conducted over 45 cycles off 30 sec at 95°C, 30 seconds at the primer specific annealing temperature (metallothionein-2 = 58°C and β-actin = 55°C) and concluded by melting curve analysis to ensure to ensure the presence of a single major amplification product. Dilution of purified plasmid stocks were used as standards for quantification. Analysis of all samples was conducted in triplicate to provide a technical validation for the quantification. Data were compared with expression levels for the same genes measured through microarray analysis. Within experiments, treatments were compared for significant differences using Student's t-test.

## Abbreviations

EST: expressed sequence tag; Cd: Cadmium; Cu: Copper; Zn: Zinc; Fe: Iron; PAH: polycyclic aromatic hydrocarbon; FLA: fluoranthene; ATZ: atrazine; AhR: Aryl hydrocarbon receptor; GO: Gene ontology; HCC:  hierarchal condition clustering; PCA: principal component analysis; ROM: reactive oxygen metabolite; ROS:  reactive oxygen species; GST: glutathione-S transferase; Q-RT-PCR: quantitative reverse transcriptase PCR.

## Authors' contributions

JO and SRS constructed earthworm cDNA libraries and sequenced ESTs, JO fabricated and hybridised the microarrays. BAH performed EST sequence analysis, established and maintained LumbriBASE. CS, PKH, MJJ and DJS generated the earthworm samples with accompanying full life history metadata and population modelling. LJL performed inorganic residue analysis. JW aided with microarray experimentation. PK was responsible for analysis of microarray data and mode of action analysis of the transcript data. DJS, CS, SRS, AJM, PK and MLB participated in conception, design and interpretation of the study. PK and MLB drafted the manuscript and together with DJS, AJM, CS, SRS, contributed to the iterative refinement of the article. All authors have read and approved the submitted version.

## Supplementary Material

Additional File 1 Table showing cDNA libraries sampled for expressed sequence tags. A summary of the cDNA libraries constructed and sampled for the project. Click here for file

Additional File 2 Sequences of oligonucleotides forming the reference probe. This table gives the sequences of the oligonucleotides used as a reference probe for the microarray hybridisation experiments. Click here for file

Additional File 3 Assessment of micro-array sensitivity and signal linearity. Representative analysis of the fluorescent signal generated by 10 RNAs introduced at known concentrations prior to labelling and detected by complementary reporter (10 replicates of each reporter spotted on the array). Panel A are data generated from Cadmium control array replicate 4, panel B is from Fluoranthene control replicate 7 and panel C is from Atrazine control replicate 1. The average signal is indicted by closed circles with technical error bars representing the standard error of the measurements. A fitted regression line is shown for the linear portion of the response together with the R^2 ^value for the fitted line. Click here for file

Additional File 4 Distribution of array micro-array data post normalisation. The distribution of the normalised data is shown for samples employed for analysing transcript changes in response to developmental stage (Panel A), together with Cadmium (Panel B) fluoranthene (Panel C), and Atrazine (Panel D) exposure. Boxes are waisted at the distribution median and encompass the interquartile range, with whiskers indicating a further 1.5× the interquartile distance. Click here for file

Additional File 5 Graphical representations of relative gene expression against fluorescence intensity from control and xenobiotic-exposed samples. Array data were normalised and filtered (as described in Materials and Methods) and the log_2 _of the average fold change (M) plotted against the log_2 _of the average mean signal intensity (A). Panel A shows a MA plot for control earthworms from the Cadmium exposure experiment whilst Panel B displays data from organisms exposed to 500 ppm Cadmium. Metallothionein ESTs are highlighted within the dashed circle. Click here for file

Additional File 6 Transcripts significantly changed between adult and juvenile earthworms. A table showing all transcripts identified as changing significantly between adult and juvenile earthworms (T-test, p < 0.01, Bonferroni False Discovery Rate) Click here for file

Additional File 7 Transcripts significantly changed in adult earthworms exposed to cadmium. A table showing all transcripts identified as changing significantly in adult earthworms exposed to cadmium (ANOVA, p < 0.05, Benjamini and Hochberg False Discovery Rate) Click here for file

Additional File 8 Transcripts significantly changed in adult earthworms exposed to fluoranthene. A table showing all transcripts identified as changing significantly in adult earthworms exposed to fluoranthene (ANOVA, p < 0.05, Benjamini and Hochberg False Discovery Rate) Click here for file

Additional File 9 Transcripts significantly changed in adult earthworms exposed to atrazine. A table showing all transcripts identified as changing significantly in adult earthworms exposed to atrazine (ANOVA, p < 0.05, Benjamini and Hochberg False Discovery Rate) Click here for file

Additional File 10 Functional Transcript Clusters Significantly (p < 0.05) Changed During Cadmium Exposure of Adult Earthworms. A table showing the functional identifications of transcripts showing altered abundance following cadmium exposure. Click here for file

Additional File 11 Functional Transcript Clusters Significantly (p < 0.05) Changed During Fluoranthene Exposure of Adult Earthworms. A table showing the functional identifications of transcripts showing altered abundance following fluoranthene exposure. Click here for file

Additional File 12 Functional Transcript Clusters Significantly (p < 0.05) Changed During Atrazine Exposure of Adult Earthworms. A table showing the functional identifications of transcripts showing altered abundance following atrazine exposure Click here for file
