# Supplementary material for: Transcriptome profiling of developmental and xenobiotic responses in a keystone soil animal, the oligochaete annelid Lumbricus rubellus
Source: BMC Genomics. 2008 Jun 3;9:266. doi: 10.1186/1471-2164-9-266 (PMC2440553; doi:10.1186/1471-2164-9-266)
Supplement: Additional File 1 — Table showing cDNA libraries sampled for expressed sequence tags. A summary of the cDNA libraries constructed and sampled for the project. [file 1471-2164-9-266-S1.doc]

**Additional File 1: The *Lumbricus rubellus*** cDNA library resource

| **Name** | **Abbreviation** | **Source of RNA** | **Vector (and 5’ cloning site)** | **Approximate Titre** | **Median size of inserts (bp)** |
| --- | --- | --- | --- | --- | --- |
| 1: Adult | Ade | Adult worms collected from a control field site and acclimatised to test condition in the laboratory | pBK-CMV  (EcoRI) | 0.5x10^6^ | 1400 |
| 2: Late Cocoon | Lc | Late developmental stage (~ 5 weeks post laying) embryonic tissue dissected from cocoons laid by paired unexposed adult worms | pBluescript II SK+ (EcoRI) | >0.5x10^6^ | 800 |
| 3: Juvenile | Jv | ~40 day post hatch juveniles (at mid log growth phase = approximately 300 mg) reared from hatchling emerging from cocoons laid by paired unexposed adult worms | pBluescript II SK+ (EcoRI) | >0.5x10^6^ | 1200 |
| 4: Anterior | Che | Head enriched (anterior segments 1-33) from acclimatised adult worms | pBluescript II SK+ (EcoRI) | >0.5x10^6^ | 1000 |
| 5: Cadmium | Cd | Mix of tissue from acclimatised adult worms exposed to either 50, 200 or 600 mg Cd / kg dry soil | pBluescript II SK+ (EcoRI) | >0.5x10^6^ | 800 |
| 6: Fluoranthene | FLA | Mix of tissue from acclimatised adult worms exposed to either 62, 140, 316, 711 and 1066 mg FLA / kg dry soil | pBluescript II SK+ (EcoRI) | >0.5x10^6^ | 800 |
| 7: Atrazine | ATZ | Mix of tissue from acclimatised adult worms exposed to either 12 and 35 mg ATZ / kg dry soil | pBluescript II SK+ (EcoRI) | >0.5x10^6^ | 1000 |
| 8: Copper | Cu | Mix of tissue from acclimatised adult worms exposed to either 40, 160, 460 and 480 mg Cu / kg dry soil | pBluescript II SK+ (EcoRI) | >0.5x10^6^ | 800 |
| 9:Reproduction subtraction |  | Reproductive organs (following a subtractive hybridisation protocol) | pGEMT (T/A cloning site) | >2000 | 200-500 |
