# Supplementary material for: Transcriptome profiling of developmental and xenobiotic responses in a keystone soil animal, the oligochaete annelid Lumbricus rubellus
Source: BMC Genomics. 2008 Jun 3;9:266. doi: 10.1186/1471-2164-9-266 (PMC2440553; doi:10.1186/1471-2164-9-266)
Supplement: Additional File 2 — Sequences of oligonucleotides forming the reference probe. This table gives the sequences of the oligonucleotides used as a reference probe for the microarray hybridisation experiments. [file 1471-2164-9-266-S2.doc]

**Additional File 2: Sequences of oligonucleotides forming the reference probe**

| **Oligonucleotide name** | **Vector from which sequence is derived** | **Libraries (see Table S1) that this oligonucleotide will hybridise to** | **Sequence** |
| --- | --- | --- | --- |
| pGEMFor | PGEMT | Libraries 1-8: Adult, Juvenile, Late Cocoon, Head Enriched, Cadmium, Fluoranthene, Atrazine, Copper | 5′ Cy3-GCCAGTGAATTGTAATACGACTCACTATAGGGCGA ATTGGGCCCGACGTCGCATGCTCCCGGCC 3′ |
| BlueScriptF | pBluescript | Library 9: Reproductive subtraction | 5′ Cy3-ACGACGGCCAGTGAGCGCGCGTAATACGACTCACT ATAGGGCGAATTGGGTACCGGGCCCCCCCTCGAG 3′ |
