# Supplementary material for: Transcriptome profiling of developmental and xenobiotic responses in a keystone soil animal, the oligochaete annelid Lumbricus rubellus
Source: BMC Genomics. 2008 Jun 3;9:266. doi: 10.1186/1471-2164-9-266 (PMC2440553; doi:10.1186/1471-2164-9-266)
Supplement: Additional File 3 — Assessment of micro-array sensitivity and signal linearity. Representative analysis of the fluorescent signal generated by 10 RNAs introduced at known concentrations prior to labelling and detected by complementary reporter (10 replicates of each reporter spotted on the array). Panel A are data generated from Cadmium control array replicate 4, panel B is from Fluoranthene control replicate 7 and panel C is from Atrazine control replicate 1. The average signal is indicted by closed circles with technical error bars representing the standard error of the measurements. A fitted regression line is shown for the linear portion of the response together with the R2 value for the fitted line. [file 1471-2164-9-266-S3.ppt]

## Slide 1
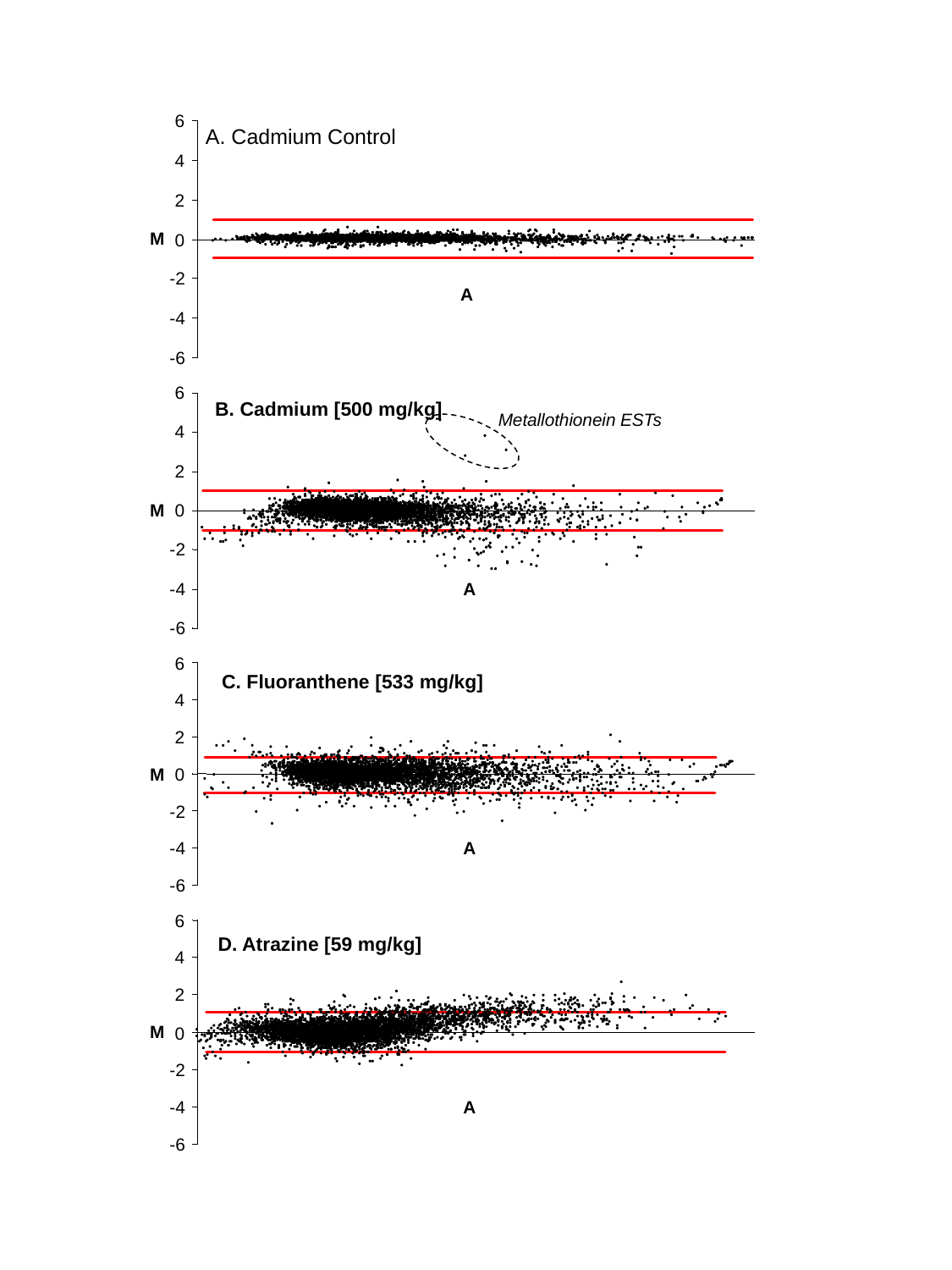

6
4
2
M
0
-2
A
-4
-6
A. Cadmium Control
6
M
4
2
0
-2
-4
A
-6
B. Cadmium [500 mg/kg]
Metallothionein ESTs
6
C. Fluoranthene [533 mg/kg]
4
2
M
0
-2
-4
A
-6
6
D. Atrazine [59 mg/kg]
4
2
M
0
-2
-4
A
-6
