# Supplementary material for: Transcriptome profiling of developmental and xenobiotic responses in a keystone soil animal, the oligochaete annelid Lumbricus rubellus
Source: BMC Genomics. 2008 Jun 3;9:266. doi: 10.1186/1471-2164-9-266 (PMC2440553; doi:10.1186/1471-2164-9-266)
Supplement: Additional File 4 — Distribution of array micro-array data post normalisation. The distribution of the normalised data is shown for samples employed for analysing transcript changes in response to developmental stage (Panel A), together with Cadmium (Panel B) fluoranthene (Panel C), and Atrazine (Panel D) exposure. Boxes are waisted at the distribution median and encompass the interquartile range, with whiskers indicating a further 1.5× the interquartile distance. [file 1471-2164-9-266-S4.ppt]

## Slide 1
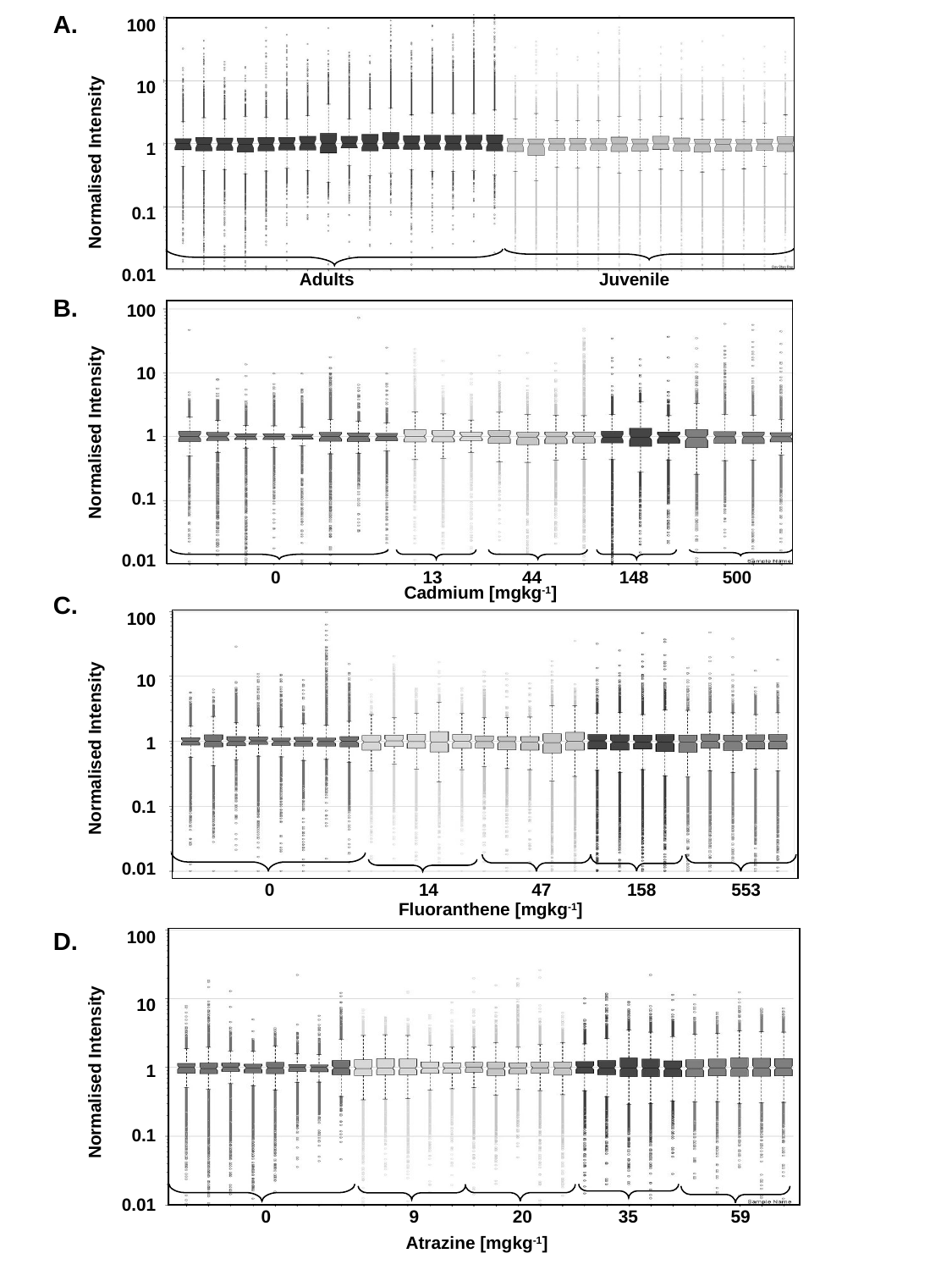

A.
100
10
1
Normalised Intensity
0.1
0.01
Adults
Juvenile
B.
100
10
Normalised Intensity
1
0.1
0.01
0
13
44
148
500
Cadmium [mgkg-1]
C.
100
10
1
Normalised Intensity
0.1
0.01
0
14
47
158
553
Fluoranthene [mgkg-1]
D.
100
10
1
Normalised Intensity
0.1
0.01
0
9
20
35
59
Atrazine [mgkg-1]
